# Blue sky as a protective factor for cardiovascular disease

**DOI:** 10.3389/fpubh.2022.1016853

**Published:** 2022-10-14

**Authors:** Haosu Tang, Congyi Zheng, Xue Cao, Su Wang, Linfeng Zhang, Xin Wang, Zuo Chen, Yuxin Song, Chen Chen, Yixin Tian, Wenping Jiang, Gang Huang, Zengwu Wang

**Affiliations:** ^1^State Key Laboratory of Numerical Modeling for Atmospheric Sciences and Geophysical Fluid Dynamics (LASG), Institute of Atmospheric Physics, Chinese Academy of Sciences, Beijing, China; ^2^Laboratory for Regional Oceanography and Numerical Modeling, Qingdao National Laboratory for Marine Science and Technology, Qingdao, China; ^3^University of Chinese Academy of Sciences, Beijing, China; ^4^State Key Laboratory of Cardiovascular Disease, Division of Prevention and Community Health, National Center for Cardiovascular Disease, National Clinical Research Center of Cardiovascular Disease, Fuwai Hospital, Peking Union Medical College, Chinese Academy of Medical Sciences, Beijing, China; ^5^Key Laboratory of Marine Hazards Forecasting, Ministry of Natural Resources, College of Oceanography, Hohai University, Nanjing, China

**Keywords:** blue sky, cohort, protective factor, cardiovascular disease, air pollution

## Abstract

**Objective:**

Blue sky has been considered to boost outdoor physical activity and social interaction, ameliorate work pressure and life stress, and enhance people's sense of happiness. However, the direct association between blue sky exposure and cardiovascular disease (CVD) still lacks epidemiological evidence. In this study, we aimed to quantify their relationship via a nationwide prospective cohort in China.

**Method:**

We extracted the baseline data from the China Hypertension Survey (CHS), by enrolling 22,702 participants aged ≥ 35 years without self-reported medical history of CVD from 14 provinces of China between 2012 and 2015 and followed up from 2018 to 2019. A blue day was marked out with no rain, low cloud cover ≤ climatological mean at each station, and visibility at 2 pm ≥ 21.52 km. We calculated the number of blue days at baseline survey year to evaluate the chronic individual blue day exposure. Cox proportional hazards models were employed to calculate the multivariable-adjusted hazard ratio (HR). We implemented subgroup analyses as well to identify potential effect modifications.

**Results:**

A total of 1,096, 993, and 597 incident cases of all-cause mortality, fatal or nonfatal CVD, and stroke occurred during a median follow-up around 5 years, respectively. A 10-day increase in annual blue day exposure was associated with a 3% (95% confidence interval [CI]: 1–6%) and 7% (95% CI: 5–10%) decreased risk of fatal or nonfatal CVD and stroke, respectively. Compared with those exposed to the worst tertile of blue days at baseline, subjects who exposed to the best tertile had a 32% (95% CI: 19–43%) and 43% (95% CI: 29–55%) lower likelihood of developing fatal or nonfatal CVD and stroke, respectively. Negative consistent exposure–response relationships were generally observed between them in the restricted cubic spline model. In the stratified analyses, the cardioprotective effects of blue sky were stronger for females, rural residents, and individuals residing in heavily contaminated areas.

**Conclusion:**

This study indicates that blue sky may serve as an independent environmental protective factor against CVD, and informs future policies on fighting air pollution and protecting the blue sky in China.

## Introduction

Cardiovascular disease (CVD), including coronary heart disease (CHD) and stroke, is the leading cause of premature mortality and long-term disability worldwide according to the Global Burden of Disease (GBD) study (1). In recent decades, a growing body of literature have well-documented the behavioral risk factors (unhealthy diet, obesity, cigarette smoking, and alcohol consumption, etc.), socio-economic risk factors (such as poverty), environmental determinants (air pollution, heat wave, and residential greenness, etc.) on the mediation of cardiovascular mortality and morbidity (2). Taking into consideration the increasing numbers of CVD incidence and the epidemiological transition (i.e., from communicable to non-communicable diseases like CVD) occurring rapidly in low- and middle-income countries (LMICs) (3), novel underlying determinants of CVD risk need to be detected, especially in developing countries such as China.

Emerging evidences in recent years suggested that residential blue spaces (coast, lakes, and rivers, etc.) could be beneficial for human health and wellbeing (4–6). An equally important but barely studied blue space is blue sky, which is ubiquitous outdoor during daytime. Blue sky has long been deemed as an indispensable part of natural ecosystem and closely tied with day-to-day lives of people. In China, from “Olympic Blue” (7, 8) to “APEC Blue” (9–11) and “Parade Blue” (12), blue sky drew widespread attention whenever mega national event was held. The number of days with blue sky that people could access dramatically affects the mental and physical health and sense of happiness of people. Up to now, whether and to what extent individual exposure to blue sky might be causally associated with beneficial health outcomes remain a knowledge gap.

During 2013–2017 and 2018–2020, Chinese government launched the first 5-year Air Pollution Prevention and Control Action Plan [APPCAP; (13)] and Three-year Action Plan to Win the Blue-Sky Defense War (14), respectively. As a result, the daily average concentrations of ambient air pollutants observed a rapid decline in China during the last decade (15, 16), together with increments in days with blue sky (17). Hence, the quantitative evaluation of the protective effects of blue sky may also carry potential policy implications and facilitate future cost/health-benefit analysis in China.

In this study, we used a population-based, nationwide, prospective cohort in China and selected three health outcomes (i.e., all-cause mortality, fatal or nonfatal CVD, and fatal or nonfatal stroke) as the endpoints. Our primary objective was to explore whether blue sky is an independent environmental protective factor for CVD, and if any, what is the quantitative relationship between them.

## Materials and methods

### Study design and population

**Figure 1:**
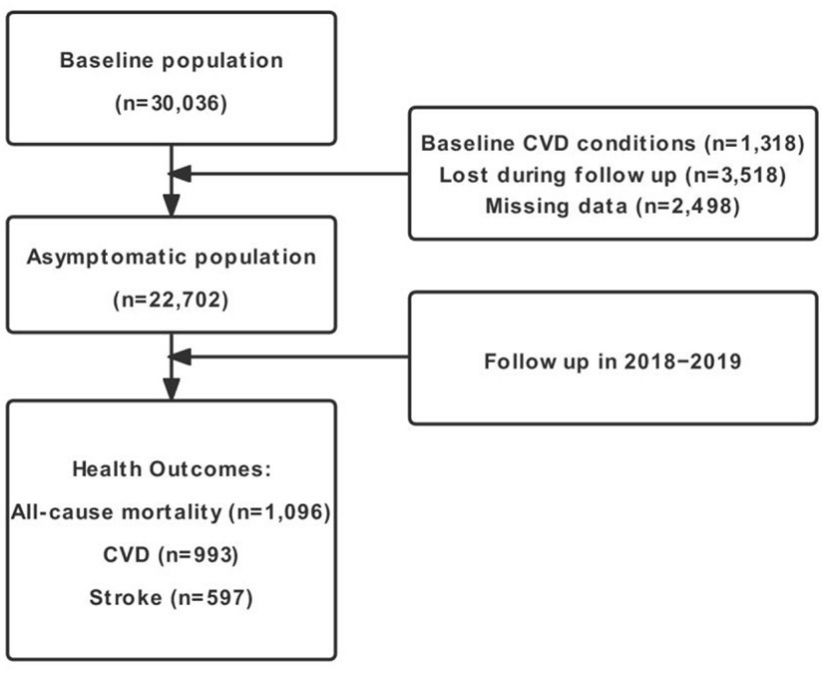
Flow diagram of study inclusion. CVD, cardiovascular disease.

The China Hypertension Survey (CHS) covered a nationwide representative sample of Chinese population *via* a 4-stage stratified multistage random sampling strategy. Details of the CHS were described in our prior studies (18, 19). In brief, during baseline from October 2012 to December 2015, the CHS surveyed ~ 500,000 participants aged ≥ 15 years from 31 provinces in China. In this study, 35,000 subjects aged ≥ 35 years from 14 provinces were further randomly selected based on their residential districts' geographical locations (East, Central, or West China) and economic development conditions (20). During follow-up between 2018 and 2019, 30,036 participants who finished the physical examinations during baseline were surveyed once again about all-cause mortality and cardiovascular events through face-to-face interview or through electronic questionnaires or telephone [median follow-up ~5 years; (21, 22)]. We excluded subjects who possessed self-reported medical history of CVD at baseline (*N* = 1,318), lost during follow-up (*N* = 3,518), and had missing values on important risk factors at baseline year (*N* = 2,498). Eventually, we included 22,702 participants overall in the formal analysis and identified no substantial differences between these two sets of study enrollees (Supplementary Table S1). Among the included subjects, three health outcomes, namely, all-cause mortality, fatal or nonfatal CVD, and fatal or nonfatal stroke were examined during follow-up. Finally, we identified 1,096, 993, and 597 incident cases of all-cause mortality, CVD, and stroke, respectively (Figure 1). This study was approved by the Ethics Committee of Fuwai Hospital (Beijing, China), and each participant offered the written informed consent prior to data collection.

### Blue day exposure assessment

The current gap in the epidemiological evidence pertaining to the effects of blue sky on CVD primarily originates from lacking in measurement techniques of blue sky. The blue sky could be either defined by ambient air quality from the perspective of environment, or assessed visually *via* cloud cover in the view of meteorology. Thus, it is determined by both environmental and large-scale meteorological conditions (23). For example, blue sky fades to pale when airborne hazes accumulate. Besides, blue sky could not appear in a day with precipitation or high percent of cloud cover even if the ambient air quality is excellent. A day with blue sky could be defined as a blue day, and here we proposed a set of standards to judge whether a day was a blue day. First, a blue day should be sunny, which could be measured by precipitation and low cloud cover. Second, a blue day should possess good air quality, which could be represented by atmospheric visibility.

We extracted observed homogenized daily mean precipitation, low cloud cover, and atmospheric visibility (recorded per day at 2 pm Beijing Time) at 378 ground meteorological stations across China during 1980–2018 after strict quality control (Supplementary Figure S1). Here, we raised three criteria for a blue day as follows: (1) no precipitation (daily precipitation ≤ 0.1 mm), (2) low cloud cover ≤ 1981–2018 climatology at each station, and (3) atmospheric visibility at 2 pm ≥ 21.52 km, which is the mean visibility at all stations during 1980–2018 in China. If a day meets the above three thresholds simultaneously, it would be considered as a blue day. This set of standards could capture almost all well-known blue events in China (APEC Blue, etc.) (24). We calculated the number of blue days at baseline survey year as the measurement of blue day exposure. Therefore, higher value of blue day index indicates more blue day exposure at baseline. We first interpolated the daily meteorological station datasets into 1 × 1 km grid via Cressman interpolation method. Afterwards, we assigned the address-based blue day exposure of each individual by averaging four grid points' values within its boundary. The impact of the definition of blue day index on the results would be discussed later in the sensitivity analysis sub-section.

### Health outcomes

Based on International Classification of Diseases−10th Revision (ICD−10), causes of death and fatal and non-fatal CVD events were coded by well-trained medical staffs at each survey site. These results were further validated *via* death certificates or medical records by the endpoint assessment committee of Fuwai Hospital (Beijing, China). In this study, CVD consisted of stroke (ICD−10 code: I60–I61 and I63–I64), coronary heart disease (I20–I25), chronic heart failure (I50), and deaths owing to CVD (I00–I25, I27–I88, and I95–99). We combined the fatal and nonfatal CVD events into the same category, which provided us relatively more cases to analyze, thereby borrowing us enhanced statistical power. Similarly, the fatal (i.e., ischemic stroke, subarachnoid hemorrhage, intracerebral hemorrhage, and unspecified stroke) and nonfatal stroke were combined as well.

### Covariates measurement

At baseline, individuals were surveyed about their demographic characteristics, including age, gender, urban/rural residence, ethnicity (Han or ethnic minorities), geographic region (East, Central, or West China), and education level. Besides, the clinical characteristics of participants were collected as well, including smoking status (current, former, or never), alcohol consumption (Yes or No), body mass index (BMI) (< 18 kg/m^2^, normal; 18–24 kg/m^2^, overweight; and > 24 kg/m^2^, obesity), hypertension, hypercholesterolemia, diabetes mellitus, family history of CVD, and CVD medication history.

The annual mean concentrations of ambient fine particulate matter of diameter ≤ 2.5 μm (PM_2.5_), nitrogen dioxide (NO_2_), and ozone (O_3_) at 15 km × 15 km resolution were derived from assimilations of surface observations from China National Environmental Monitoring Center (25). Aided by the chemical data assimilation system (ChemDAS) developed by the Institute of Atmospheric Physics (Beijing, China), these assimilation products show relatively high consistency with independent observations (*R*^2^ = 0.74–0.86). The baseline ambient air pollutant exposures were assigned based on each participant's residential address. Besides, we obtained annual mean ambient temperature, maximum temperature, relative humidity, and altitude from ~2419 meteorological stations across China to denote large-scale meteorological conditions and assigned these variables likewise (Supplementary Figure S2). These meteorological datasets were strictly quality controlled by the National Meteorological Information Center (NMIC) of China Meteorological Administration (CMA) (http://data.cma.cn/).

Moreover, annual average years of education, per capita gross domestic product (GDP), and population density across survey counties were extracted from the Data Center for Resources and Environmental Sciences for China (https://www.resdc.cn/) as surrogates to demonstrate socioeconomic statuses. We also derived normalized difference vegetation index (NDVI) and enhanced vegetation index (EVI) with high spatial (1 km × 1 km) and temporal (monthly) resolutions from the moderate resolution imaging spectroradiometer (MODIS) products (MOD13A3, version 6, https://lpdaacsvc.cr.usgs.gov/appeears/), which has been widely used to denote the long-term residential greenness exposure. In addition, we calculated the frequency of extreme heat waves to denote the extent of sun exposure, which is defined as annual number of days when maximum temperature ≥ 90th percentile of 1961–1990 climatology (26). Likewise, we assigned these variables to each participant according to their geocoded residential address.

### Statistical analysis

We presented the continuous variables with means ± standard deviation (SD), while the categorical variables with absolute numbers (relative percentages). The between-group comparisons of continuous (categorical) variables were examined *via* *ANOVA* (χ^2^) test. We calculated the incidence rates (per 1,000 person-years) of three health outcomes. Cox proportional hazards models were employed to quantify longitudinal association between blue day exposure and all-cause mortality and cardiovascular events. We interpreted the derived results *via* two methods. First, we used a 10-day increment in the annual blue day exposure. Besides, we categorized the blue day exposure into three categories (worst tertile [ ≤ 42.0 days/year], middle tertile [42.0–77.4 days/year], best tertile [≥ 77.4 days/year]). Hazard ratios (HRs) were shown with the worst tertile as the reference group. We first applied the change in estimate approaches combined with directed acyclic graphs (DAG) to select the confounders. Finally, we adjusted for the following lists of covariates: (a) In model 1, we adjusted for age, sex, urbanity, ethnicity, geographic region (East, Central, and West), educational level, smoking status, alcohol consumption, and BMI; (b) In model 2, we additionally adjusted for hypertension, hypercholesterolemia, diabetes mellitus, family history of CVD, CVD medication history; (c) In model 3, we additionally adjusted for outdoor PM_2.5_ concentrations and ambient temperature; (d) In model 4, we further adjusted for county-level average years of education, per capita GDP, population density and altitude.

Given the possible nonlinear association between chronic blue day exposure and all-cause mortality and cardiovascular events, we included the restricted cubic spline (RCS) model in our Cox model. The knot number was determined by Akaike information criterion (AIC), while the nonlinear association was examined via the likelihood ratio test. Point estimates and 95% confidence intervals (CIs) reporting as the 2.5th and 97.5th percentiles of the posterior distributions were calculated. Finally, we implemented a series of subgroup analyses, stratified by age (35–59, ≥ 60 years old), sex, urbanity, BMI (< 24, ≥ 24 kg/m^2^), outdoor PM_2.5_ concentrations (< 60, ≥ 60 μg/m^3^), and individual medical history (i.e., hypertension, hypercholesterolemia, and diabetes mellitus).

To test the robustness of the results in the main analyses, several sensitivity analyses were performed: (a) using another definition of blue day index, which was based on fixed threshold values (22); (b) using another window of blue day exposure (i.e., 3-year mean before baseline year); (c) changing outdoor PM_2.5_ concentration into dichotomous variable given the possible collinearity between blue day index and PM_2.5_; (d) removing participants who died or got disease within the first year after the baseline survey; (e) excluding subjects whose residential address changed during follow-up to evaluate the possible impact of the movement of study participants; (f) excluding subjects who possessed baseline life-limiting chronic diseases (i.e., tumor, kidney disease, chronic obstructive pulmonary disease, and rheumatic immune disease) which could likely be fatal within the next few years; (g) including relative humidity at baseline year additionally as the covariate; (h) including frequency of extreme heat waves additionally as the covariate; (i) including NDVI or EVI additionally as the covariate; (j) including NO_2_ or O_3_ additionally as the covariate.

The statistical significance was delineated at two-tailed *p* < 0.05. SAS version 9.4 (SAS Institute Inc, Cary, NC) and R software version 4.1.1 (R Foundation for Statistical Computing, Vienna, Austria) were used to conduct statistical analyses.

## Results

### Descriptive statistics

Table 1 demonstrated the descriptive statistics for the enrolled participants at baseline according to blue day exposure tertile. Our CHS cohort included 22,702 entrants, of which 46.3% were males. Among the three groups, participants with higher blue day exposure tended to be less educated and have lower BMI. Moreover, they had a higher prevalence of hypercholesterolemia and family history of CVD, and were less exposed to PM_2.5_ in general.

**Table 1:** Baseline characteristics of study participants.

**Characteristics**	**Overall (*n =* 22,702)**	**Tertiles of blue days (days/year)**			***P* value**
		**Worst tertile**	**Middle tertile**	**Best tertile**	
		**3.7–42.0 (*n =* 7,426)**	**42.0–77.4 (*n =* 6,962)**	**77.4–122.3 (*n =* 8,314)**	
Age, years	56.1 ± 13.1	56.2 ± 13.4	57.5 ± 12.4	54.9 ± 13.3	<.001
Male	10,505 (46.3)	3,512 (47.3)	2,946 (42.3)	4,047 (48.7)	<.001
Urban	10,130 (44.6)	4,090 (55.1)	2,530 (36.3)	3,510 (42.2)	<.001
Han ethnicity	20,315 (89.5)	7,360 (99.1)	5,342 (76.7)	7,613 (91.6)	<.001
Region					<.001
East	9,263 (40.8)	3,167 (42.6)	2,120 (30.5)	3,976 (47.8)	
Central	9,460 (41.7)	4,259 (57.4)	1,899 (27.3)	3,302 (39.7)	
West	3,979 (17.5)	0 (0)	2,943 (42.2)	1,036 (12.5)	
Education at least middle school	11,109 (48.9)	3,783 (50.9)	3,348 (48.1)	3,978 (47.8)	<.001
Smoking					<.001
Current	5,731 (25.2)	1,878 (25.3)	1,582 (22.7)	2,271 (27.3)	
Former	1,216 (5.4)	313 (4.2)	450 (6.5)	453 (5.4)	
Never	15,755 (69.4)	5,235 (70.5)	4,930 (70.8)	5,590 (67.2)	
Alcohol consumption	6,318 (27.8)	1,893 (25.5)	1,743 (25.0)	2,682 (32.3)	<.001
BMI (kg/m^2^)					<.001
Normal	10,213 (45.0)	3,156 (42.5)	2,994 (43.0)	4,063 (48.9)	
Overweight	8,533 (37.6)	2,891 (38.9)	2,663 (38.3)	2,979 (35.8)	
Obesity	3,956 (17.4)	1,379 (18.6)	1,305 (18.7)	1,272 (15.3)	
Hypertension	8,957 (39.5)	2,914 (39.2)	3,044 (43.7)	2,999 (36.1)	<.001
Hypercholesterolemia	7,724 (34.0)	2,221 (29.9)	2,488 (35.7)	3,015 (36.3)	<.001
Diabetes mellitus	2,286 (10.1)	706 (9.5)	840 (12.1)	740 (8.9)	<.001
Family history of CVD	2,621 (11.5)	793 (10.7)	791 (11.4)	1,037 (12.5)	<.001
CVD medication history	4,929 (21.7)	1,577 (21.2)	1,892 (27.2)	1,460 (17.6)	<.001
Ambient temperature, (°C)	24.6 ± 4.3	26.8 ± 2.4	22.2 ± 5.7	24.7 ± 3.1	<.001
PM_2.5_, (μg/m^3^)	61.7 ± 22.5	70.8 ± 25.2	59.0 ± 23.9	55.9 ± 15.1	<.001
NO_2_, (μg/m^3^)	29.3 ± 13.3	33.7 ± 11.1	26.8 ± 18.4	27.4 ± 8.1	<.001
O_3_, (μg/m^3^)	56.7 ± 10.4	56.7 ± 10.7	57.1 ± 10.8	56.3 ± 9.8	<.001

Numbers are mean ± SD or no. (%). BMI, body mass index; CVD, cardiovascular disease; PM_2.5_, particles with an aerodynamic diameter of ≤ 2.5 μm; NO_2_, nitrogen dioxide; O_3_, ozone.

**Figure 2:**
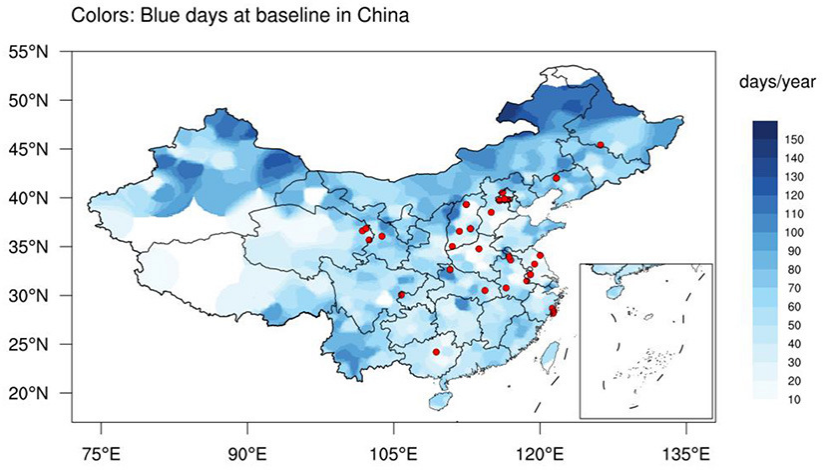
The geographical distributions of annual blue days (color, days/year) and 30 China Hypertension Survey sites (red dots) at baseline (2012–2015) in China.

Figure 2 described the geographical distributions of annual blue days at baseline survey year in China. Vast urban agglomerations in China (Beijing-Tianjin-Hebei region and the Yangtze River Delta, etc.) had relatively few blue days for the period 2012–2015, possibly due to severe air pollution induced by rapid land urbanization and unoptimized energy structure (27). On the other side, Northeast China, Inner Mongolia, North Xinjiang, and Yunnan province had a great many blue days as high as 100 days/year, probably thanks to favorable weather conditions all year round. We further calculated the stratified numbers of baseline annual blue days (Supplementary Table S2). The results showed that rural residents and people living in west China tended to enjoy more blue days than urban counterparts and those in east and central China, respectively. Besides, higher exposure to blue days was generally associated with lower ambient mean temperature. We also noted that lower PM_2.5_ or NO_2_ did not necessarily denote more blue day exposure, which could be due to the modification by large-scale meteorological conditions. We calculated the Pearson correlation coefficients between PM_2.5_ or NO_2_ and blue day index for all counties/districts, and the correlations are nonsignificant.

### Associations of blue sky with health outcomes

**Figure 3:**
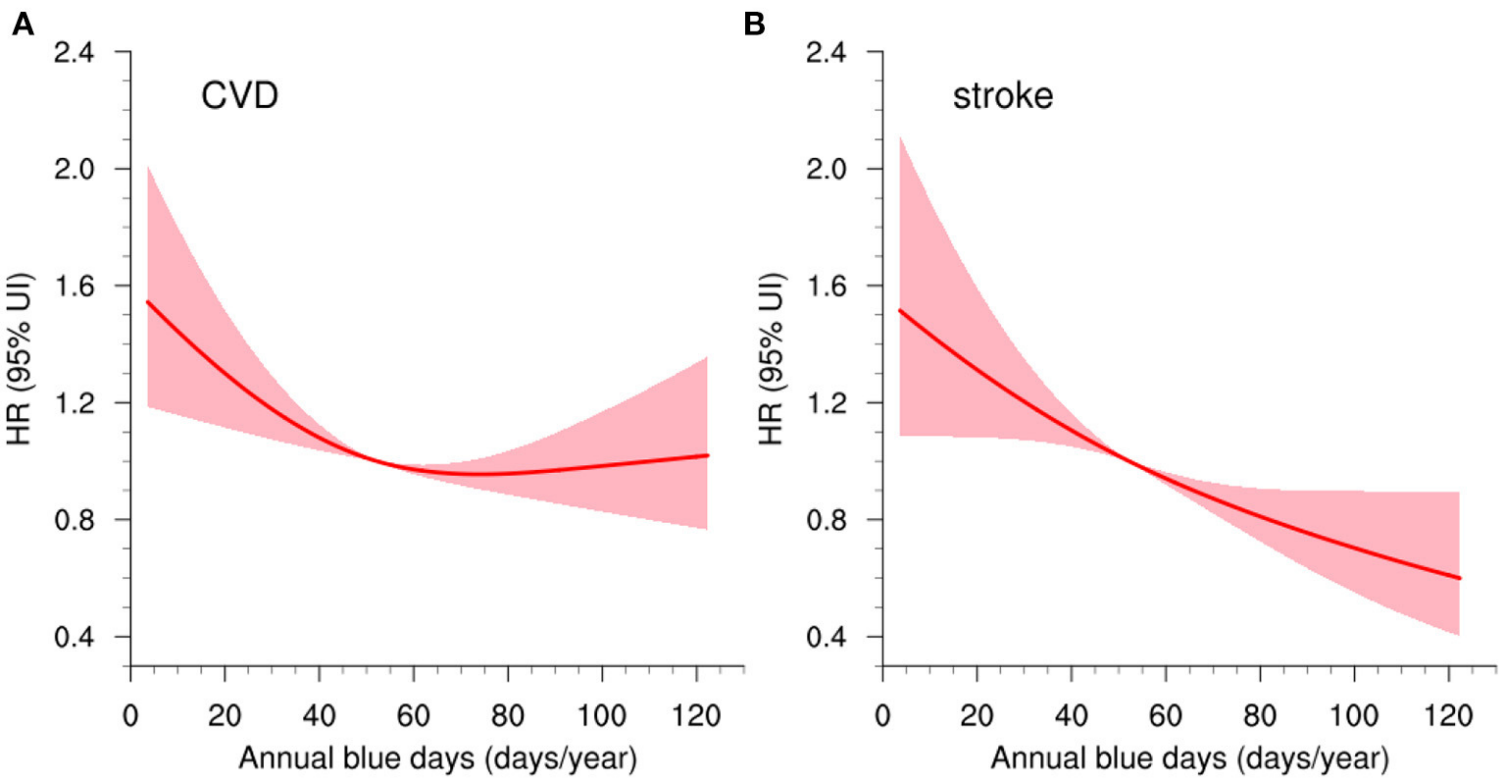
Concentration–response curves (red lines) with 95% CIs (shadings) of blue day exposure (days/year) and **(A)** fatal or nonfatal CVD, and **(B)** fatal or nonfatal stroke. CVD, cardiovascular disease.

At ~5-year follow-up, the incidence rates of all-cause mortality, fatal or nonfatal CVD, and stroke were 10.49, 9.45, and 5.64 per 1,000 person-years, respectively. In the multivariable-adjusted Cox regression models, a 10-day increase in annual blue day exposure was associated with 3% (95% CI: 1–6%) and 7% (95% CI: 5–10%) lower risks of fatal or nonfatal CVD and stroke, respectively (Table 2). It suggested that blue sky may serve as the protective factor for cardiovascular events. In comparison with those exposed to the worst tertile of blue days at baseline, subjects who exposed to the best tertile had a 32% (95% CI: 19–43%, *p*_*trend*_ < 0.001) and 43% (95% CI: 29–55%, *p*_*trend*_ < 0.001) lower likelihood of developing fatal or nonfatal CVDs and strokes, respectively. The association in all-cause mortality was not significant (HR = 1.00, 95% CI: 0.98–1.02). Negative consistent dose-response relationships were observed in blue day exposure and fatal or nonfatal CVD and stroke as well (Figure 3). The associations between blue day exposure and the HRs were L-shaped for CVD (*p*_*nonlinearity*_ < 0.05) and linear-shaped for stroke (*p*_*nonlinearity*_ = 0.083).

**Table 2:** Hazard ratios (95% CIs) for chronic blue day exposure with all-cause mortality and cardiovascular events.

	**Per 10-day increment**	**Tertiles of blue days**			***P*_trend_**
		**Worst tertile**	**Middle tertile**	**Best tertile**	
**All-cause mortality**					
No. of cases	1,096	320	347	429	/
Incidence rate^†^	10.49	10.25	10.73	10.50	/
Adjusted Model 1^a^	1.00 (0.98–1.02)	1.00 (ref)	0.89 (0.74–1.06)	0.93 (0.80–1.08)	0.421
Adjusted Model 2^b^	1.00 (0.98–1.02)	1.00 (ref)	0.86 (0.72–1.03)	0.93 (0.80–1.08)	0.455
Adjusted Model 3^c^	1.00 (0.97–1.02)	1.00 (ref)	0.80 (0.66–0.96)	0.85 (0.72–1.00)	0.131
Adjusted Model 4^d^	1.00 (0.98–1.02)	1.00 (ref)	0.84 (0.70–1.00)	0.88 (0.75–1.04)	0.241
**CVD (fatal** **+** **nonfatal)**					
No. of cases	993	305	347	341	/
Incidence rate^†^	9.45	9.73	10.69	8.27	/
Adjusted Model 1^a^	0.98 (0.96–1.00)	1.00 (ref)	1.13 (0.95–1.34)	0.80 (0.68–0.93)	0.002
Adjusted Model 2^b^	0.98 (0.96–1.00)	1.00 (ref)	1.06 (0.90–1.26)	0.78 (0.67–0.92)	0.001
Adjusted Model 3^c^	0.96 (0.94–0.98)	1.00 (ref)	0.91 (0.76–1.10)	0.63 (0.53–0.76)	<.001
Adjusted Model 4^d^	0.97 (0.94–0.99)	1.00 (ref)	0.86 (0.72–1.03)	0.68 (0.57–0.81)	<.001
**Stroke (fatal** **+** **nonfatal)**					
No. of cases	597	192	209	196	/
Incidence rate^†^	5.64	6.09	6.39	4.72	/
Adjusted Model 1^a^	0.95 (0.92–0.97)	1.00 (ref)	1.19 (0.95–1.49)	0.70 (0.57–0.86)	<.001
Adjusted Model 2^b^	0.94 (0.92–0.97)	1.00 (ref)	1.11 (0.89–1.40)	0.69 (0.56–0.84)	<.001
Adjusted Model 3^c^	0.91 (0.88–0.94)	1.00 (ref)	0.93 (0.74–1.17)	0.50 (0.39–0.64)	<.001
Adjusted Model 4^d^	0.93 (0.90–0.95)	1.00 (ref)	0.80 (0.64–1.00)	0.57 (0.45–0.71)	<.001

^†^Incident rate per 1,000 person-years.

CVD, cardiovascular disease.

^a^Model 1: adjusted for age, sex, urbanity, ethnicity, geographic region, educational level, smoking status, alcohol consumption, and BMI.

^b^Model 2: Model 1 + hypertension, hypercholesterolemia, diabetes mellitus, family history of CVD, and CVD medication history.

^c^Model 3: Model 2 + outdoor PM_2.5_ concentrations, and ambient temperature.

^d^Model 4: Model 3 + county-level average years of education, per capita GDP, population density, and altitude.

**Figure 4:**
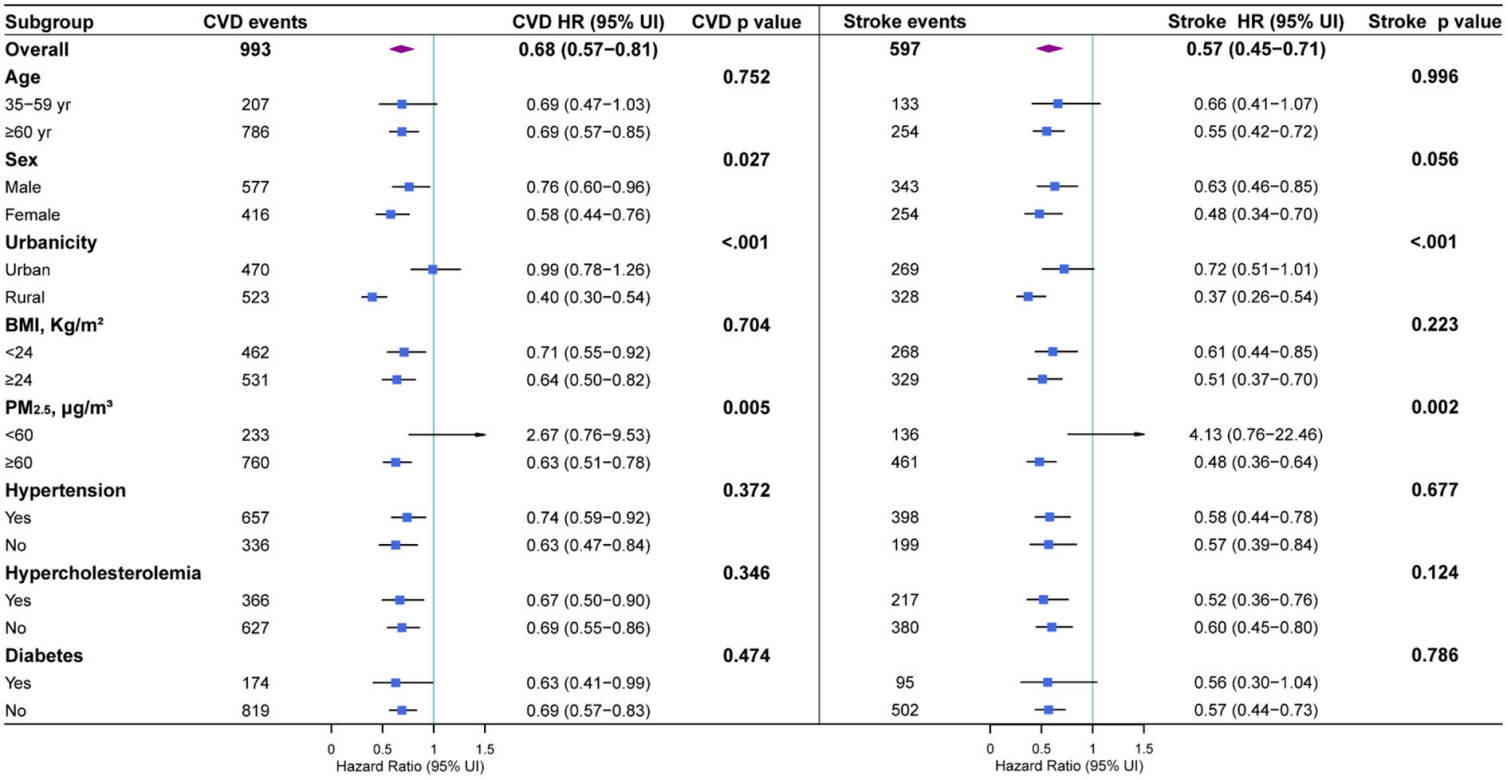
Subgroup analyses for full-adjusted hazard ratios (95% CIs) of fatal or nonfatal CVD and stroke with the best tertile compared to the worst tertile in annual blue day exposure.

Figure 4 presented the subgroup analysis results as well as interaction tests to assess the underlying effect modifications. In sex-stratified subgroup, females were inclined to benefit more from blue day exposure than males. When classifying by urbanicity, we found a significant protection effect only in rural residents. Compared with rural residents living in the worst tertile of blue day exposure, those in the best tertile had 60% (95% CI: 46–70%, *p*_*interaction*_ < 0.001) lower odds of developing CVDs, and 63% (95% CI: 46–74%, *p*_*interaction*_ < 0.001) fewer strokes. Moreover, a more pronounced protection effect was observed in people living in heavily contaminated areas (PM_2.5_ ≥ 60 μg/m^3^), with 37% (95% CI: 22–49%, *p*_*interaction*_ = 0.005) fewer CVDs and 52% (95% CI: 36–64%, *p*_*interaction*_ < 0.005) fewer strokes in the best tertile compared with the worst one.

### Sensitivity analyses

The results in the main analysis were consistently observed in almost all sensitivity analyses (Supplementary Table S3), implying that different definitions of blue day index, exposure windows, groups of study enrollees, and further adjustments for more underlying covariates did not substantially bias the main conclusions.

## Discussion

This population-based nationwide prospective study investigated the relationship between chronic blue day exposure and CVD among individuals aged 35 years and older in China. This cohort investigation found that higher levels of blue day exposure were longitudinally associated with lower risks of cardiovascular incidences, especially in females, rural residents, and people living in heavily contaminated areas. These results robustly hold in the sensitivity analyses. Our work may carry potential societal advantages and public health importance. We highlighted that policies to increase blue sky in China may provide opportunities for better cardiovascular fitness.

In the present study, we observed a slightly stronger cardioprotective effect of blue sky for females. This gender disparity may be partly explained by the hypothesis that women might be more susceptible to the change of external environments (28). Besides, we found that rural residents were more likely to benefit from blue sky than their urban counterparts. It could to some extent be attributed to the outdoor farm work such as the spring planting and autumn harvest by rural residents in China. In that case, they may have more actual contact with blue sky (Supplementary Table S2). In addition, people in heavily contaminated areas were observed to gain more cardiovascular protective benefits of blue sky. This is in accord with guidelines on CVD prevention in clinical practice (29), which indicated that individuals with the highest risk at baseline tend to obtain the most benefits from preventive efforts.

Over recent decades, short- and long-term exposure to air pollutants, as typified by PM_2.5_, are suggested important modifiable risk factors of cardiovascular outcomes (morbidity, mortality and biomarkers) (30–32), and thereby have become an emerging focus field in environmental epidemiology. This study offered a different view to evaluate how the clean air would benefit CVD since the clean air contributes to blue sky. Here, we proposed several potential pathways and mechanistic links by which chronic blue day exposure could lower cardiovascular incidences. First, increased blue day exposures are often tied with lower exposure to ambient air pollutants and better air quality, which are beneficial for cardiovascular health (33–35). Second, people under higher blue day exposure are more willing to enjoy outdoor sports and have contact with natural environment. An extensive number of studies have documented the benefits of physical exercise on elevated cardiovascular fitness through adaptations to the heart and vascular system (36). Recent evidence from cardiovascular cohorts demonstrated that the cardioprotective effects of physical exercise may mediate the traditional or untraditional CVD risk factors. Specifically, regular physical activity practice is related to decreased blood pressure and arterial stiffness, increased insulin sensitivity, and a more favorable inflammatory marker profile (37). Thus, enhanced access to blue sky is associated with a better physical quality of life and fewer incidences of non-communicable diseases like CVD (38). Third, blue sky may significantly relieve feelings of anxiety and depression. This psychological recovery will contribute to lower risks of CVD (39). Fourth, blue day exposures would exert a subtle influence on forming social relationships since people are more likely to meet each other in the neighborhood, which may further promote positive outlooks on life as well as active and healthy lifestyles. This boost in social interaction and cohesion would impede the development of CVD (40).

### Implications and future research

During the past decade, growing environmental epidemiological studies focused on the blue spaces (lakes, waterways, canals, ponds, coasts, etc.), indicating that living near the coast or inland water bodies may serve as a protective factor for human health including CVD (4–6). In the present study, we attempted to extend the concept of traditional blue spaces and probed the potential relationship between blue sky exposure and CVD. The observations in this study provided a novel line of evidence from existing literatures that how the environment would mediate the burden of CVD. Moreover, we obtained a nationally representative dose–response curve for the blue sky–CVD relationship *via* the RCS model and it could be informative for policymakers to evaluate current progress toward clean air and blue sky, and guide future environmental policy-making and public health advancement in China.

However, owing to the partial nature of our work, it is still too early to provide definitive answer about the beneficial effects of blue sky on human health at this stage. Further studies are required to reveal the relationships between blue sky and other health outcomes and the underlying mechanisms. Besides, it ought to be acknowledged that the definition of blue sky in this study is subjective to some extent given the selection of the three criteria. We would leave such efforts to future studies that could define a more objective blue sky index through technologies such as the satellite remote sensing. Moreover, due to the limited access to observational meteorological, environmental, and human health records, the study region of the present study was confined in China. Future investigations on the potential health benefits of blue skies on world's population living in South Asia, Africa and South America where the air pollution risks are still heavy may be preferable. Furthermore, we have not yet analyzed the respective contribution of air pollution and meteorology/climate to the number of blue days. Additional studies can employ climate model simulations to differentiate the effects of climate from those of air pollution in space and in time (16), which would favor future cost/health-benefit analysis of air pollution reduction programs in China.

### Strength and limitations

The major strength of this study is that we identified a potential independent environmental protective factor for CVD, i.e., blue sky. Although blue sky has long been deemed beneficial for cardiovascular health intuitively, here we strived to provide epidemiological evidence and build up their statistical relationship. These results might be instrumental in the attribution of GBD from environmental causes. Besides, we adopted a nationally representative cohort and incorporated a relatively large sample size, which would elevate the statistical power of the findings in this study. Moreover, our study baseline recruitment and follow-up periods were nearly simultaneous with air pollution reduction programs in China that were implemented in 2013–2020, offering the latest high-quality cohort evidence pertaining to the CVD burden associated with the blue sky. Finally, we accounted for a variety of covariates (including demographic, clinical, environmental, and socioeconomic covariates) to adjust for the latent confounding as far as possible.

Despite these advantages, the limitations of the present study also merit consideration. First, the present study was essentially an ecological study, and the discrepancy between individual perception and environmental concentrations (i.e., exposure misclassification) may to some extent bias our results. Second, the limited follow-up period of our CHS cohort (median ~5 years) may somewhat weaken the statistical power of our results. Thus, the insignificant association between blue day exposure and all-cause mortality shown in this study does not necessarily mean there is no connection between them and we should treat this result with caution. Future researches of a longer follow-up period would be carried out as our CHS cohort marches forward. Additionally, although we adjusted for the corresponding covariates, a likelihood of residual confounding still exists. For instance, the simple “Yes or No” binary classification may be insufficient to adequately control for stressors related with alcohol consumption. A quantitative classification as (a) no alcohol consumption (never), (b) no alcohol consumption (former), (c) low consumption, and (d) high consumption based on national/international guidelines would be preferable. Therefore, our findings need to be confirmed by future studies in other settings. Last, we cannot rule out the potential confounding biases brought by other covariates such as dietary habits and exercise. These potential mediating, confounding or modifying effects may be important and deserve future studies.

## Conclusions

In summary, findings from this study offered nationally representative epidemiological evidence for a significant protective association of long-term blue sky exposure with fatal or nonfatal CVD, adjusting for demographic, clinical, environmental, and socioeconomic covariates. This study provided novel insights on probing the environmental determinants of CVD risk. In tandem with previous studies, our work appeals for the continuous implementation of air quality management in China in the future to further protect the blue sky and promote cardiovascular health.

## Data availability statement

The meteorological and environmental datasets used in this study are available from the corresponding author upon request. Requests should be directed to ZW to access the CHS cohort datasets.

## Ethics statement

The studies involving human participants were reviewed and approved by Ethics Committee of Fuwai Hospital. Written informed consent to participate in this study was provided by the participants' legal guardian/next of kin.

## Author contributions

HT: conceptualization, methodology, formal analysis, software, visualization, and writing—original draft preparation. XC: methodology, data curation, formal analysis, software, and investigation. CZ: software, investigation, and validation. GH and ZW: conceptualization and funding acquisition. All authors: writing—reviewing and editing.

## Funding

The study is jointly supported by the National Natural Science Foundation of China (42141019, 41831175, 91937302, 42175040, and 41721004), and the Youth Innovation Promotion Association of CAS (2021072).

## Conflict of interest

The authors declare that the research was conducted in the absence of any commercial or financial relationships that could be construed as a potential conflict of interest.

## Publisher's note

All claims expressed in this article are solely those of the authors and do not necessarily represent those of their affiliated organizations, or those of the publisher, the editors and the reviewers. Any product that may be evaluated in this article, or claim that may be made by its manufacturer, is not guaranteed or endorsed by the publisher.

## References

[1] Murray CJL Aravkin AY Zheng P Abbafati C Abbas KM Abbasi-Kangevari M. Global burden of 87 risk factors in 204 countries and territories, 1990–2019: a systematic analysis for the Global Burden of Disease Study (2019). Lancet. (2020) 396:1223–49. 10.1016/S0140-6736(20)30752-2 33069327 PMC7566194

[2] Joseph P Leong D McKee M Anand SS Schwalm J-D Teo K. Reducing the global burden of cardiovascular disease, part 1: the epidemiology and risk factors. Circ Res. (2017) 121:677–94. 10.1161/CIRCRESAHA.117.308903 28860318

[3] Roth GA Mensah GA Johnson CO Addolorato G Ammirati E Baddour L. Global burden of cardiovascular diseases and risk factors, 1990-2019: update from the GBD 2019 study. J Am Coll Cardiol. (2020) 76:2982–3021. 10.1016/j.jacc.2020.11.010 33309175 PMC7755038

[4] McDougall CW Quilliam RS Hanley N Oliver DM. Freshwater blue space and population health: an emerging research agenda. Sci Total Environ. (2020) 737:140196. 10.1016/j.scitotenv.2020.140196 32783838

[5] Pasanen TP White MP Wheeler BW Garrett JK Elliott LR. Neighbourhood blue space, health and wellbeing: the mediating role of different types of physical activity. Environ Int. (2019) 131:105016. 10.1016/j.envint.2019.105016 31352260

[6] Smith N Georgiou M King AC Tieges Z Webb S Chastin S. Urban blue spaces and human health: a systematic review and meta-analysis of quantitative studies. Cities. (2021) 119:103413. 10.1016/j.cities.2021.103413

[7] Schleicher N Norra S Chen Y Chai F Wang S. Efficiency of mitigation measures to re-duce particulate air pollution—a case study during the Olympic Summer Games 2008 in Beijing, China. Sci Total Environ. (2012) 427:146–58. 10.1016/j.scitotenv.2012.04.004 22560243

[8] Streets DG Fu JS Jang CJ Hao J He K Tang X. Air quality during the 2008 Beijing Olympic Games. Atmos Environ. (2007) 41:480–92. 10.1016/j.atmosenv.2006.08.046

[9] Li X Qiao Y Zhu J Shi L Wang Y. The ‘APEC blue' endeavor: causal effects of air pollution regulation on air quality in China. J Clean Prod. (2017) 168:1381–8. 10.1016/j.jclepro.2017.08.164

[10] Meng R Zhao FR Sun K Zhang R Huang C Yang J. Analysis of the 2014. ‘APEC Blue' in Beijing using more than one decade of satellite observations: lessons learned from radical emission control measures. Remote Sens. (2015) 7:15224–43. 10.3390/rs71115224

[11] Liu H He J Guo J Miao Y Yin J-F Wang Y. The blue skies in Beijing during APEC 2014: a quantitative assessment of emission control efficiency and meteorological influence. Atmos Environ. (2017) 167:235–44. 10.1016/j.atmosenv.2017.08.032

[12] Xue Y Wang Y Li X Tian H Nie L Wu X. Multi-dimension apportionment of clean air ‘parade blue' phenomenon in Beijing. J Environ Sci. (2018) 65:29–42. 10.1016/j.jes.2017.03.035 29548400

[13] China State Council. Action Plan on Prevention Control of Air Pollution. (2013). Available online at: http://www.gov.cn/zwgk/2013-09/12/content_2486773.htm (accessed Septembber 15, 2022).

[14] China State Council. The Three-year Action Plan for Blue-Sky Defense War. (2018). Available online at: http://www.gov.cn/xinwen/2019-03/21/content_5375551.htm. (accessed Septembber 15, 2022).

[15] Xue T Liu J Zhang Q Geng G Zhang Y Tong D. Rapid improvement of PM2.5 pollution and associated health benefits in China during 2013–2017. Sci China Earth Sci. (2019) 62:1847–56. 10.1007/s11430-018-9348-2

[16] Zhang Q Zheng Y Tong D Shao M Wang S Zhang Y. Drivers of improved PM2.5 air quality in China from 2013 to 2017. Proc Natl Acad Sci USA. (2019) 116:24463–9. 10.1073/pnas.1907956116 31740599 PMC6900509

[17] Wang S Huang G Dai T Hu K. The first 5-year clean air action did increase the blue days in winter over Beijing-Tianjin-Hebei. Sci Bull. (2022) 67:774–6. 10.1016/j.scib.2022.01.009 36546227

[18] Hao G Wang X Chen Z Zhang L Zhang Y Wei B. Prevalence of heart failure and left ventricular dysfunction in China: the China Hypertension Survey, 2012–2015. Eur J Heart Fail. (2019) 21:1329–37. 10.1002/ejhf.1629 31746111

[19] Wang Z Chen Z Zhang L Wang X Hao G Zhang Z. Status of hypertension in China. Circulation. (2018) 137:2344–56. 10.1161/CIRCULATIONAHA.117.032380 29449338

[20] Wang Z Chen Z Wang X Zhang L Li S Tian Y. The disease burden of atrial fibrillation in China from a national cross-sectional survey. Am J Cardiol. (2018) 122:793–8. 10.1016/j.amjcard.2018.05.015 30049467

[21] Cao X Tang H Zheng C Kang Y Zhang L Wang X. Association of heating fuel types with mortality and cardiovascular events among non-smokers in China. Environ Pollut. (2021) 291:118207. 10.1016/j.envpol.2021.118207 34563845

[22] Kang Y Tang H Zhang L Wang S Wang X Chen Z. Long-term temperature variability and the incidence of cardiovascular diseases: a large, representative cohort study in China. Environ Pollut. (2021) 278:116831. 10.1016/j.envpol.2021.116831 33711625

[23] Wang S Huang G Lin J Hu K Wang L Gong H. Chinese blue days: a novel index and spatio-temporal variations. Environ Res Lett. (2019) 14:074026. 10.1088/1748-9326/ab29bb

[24] Wang S Huang G Hu K Wang L Dai T Zhou C. The deep blue day is decreasing in China. Theor Appl Climatol. (2022) 147:1–10. 10.1007/s00704-021-03898-1 35095143 PMC8782681

[25] Kong L Tang X Zhu J Wang Z Li J Wu H. A 6-year-long (2013–2018) high-resolution air quality reanalysis dataset in China based on the assimilation of surface observations from CNEMC. Earth Syst Sci Data. (2021) 13:529–70. 10.5194/essd-13-529-2021

[26] Zhang X Alexander L Hegerl GC Jones PD Tank AK Peterson TC. Indices for monitoring changes in extremes based on daily temperature and precipitation data. Wiley Interdiscip Rev: Climate Change. (2011) 2:851–70. 10.1002/wcc.147

[27] Huang C Liu K Zhou L. Spatio-temporal trends and influencing factors of PM 2.5 concentrations in urban agglomerations in China between 2000 and (2016). Environ Sci Pollut Res. (2021) 28:10988–1000. 10.1007/s11356-020-11357-z 33108644

[28] Stafford M Cummins S Macintyre S Ellaway A Marmot MG. Gender differences in the associations between health and neighbourhood environment. Soc Sci Med. (2005) 60:1681–92. 10.1016/j.socscimed.2004.08.028 15686801

[29] Piepoli MF Hoes AW Agewall S Albus C Brotons C Catapano AL. European Guidelines on cardiovascular disease prevention in clinical practice. Eur Heart J. (2016) 37:2315–81. 10.1093/eurheartj/ehw106 27222591 PMC4986030

[30] Chen J Hoek G. Long-term exposure to PM and all-cause and cause-specific mortality: a systematic review and meta-analysis. Environ Int. (2020) 143:105974. 10.1016/j.envint.2020.105974 32703584

[31] Hvidtfeldt UA Sørensen M Geels C Ketzel M Khan J Tjønneland A. Long-term residential exposure to PM2.5, PM10, black carbon, NO2, and ozone and mortality in a Danish cohort. Environ Int. (2017) 123:265–72. 10.1016/j.envint.2018.12.010 30551059

[32] Zhang Y. All-cause mortality risk and attributable deaths associated with long-term exposure to ambient PM2.5 in Chinese adults. Environ Sci Technol. (2021) 55:6116–27. 10.1021/acs.est.0c08527 33870687

[33] Liang F Liu F Huang K Yang X Li J Xiao Q. Long-term exposure to fine particulate matter and cardiovascular disease in China. J Am Coll Cardiol. (2020) 75:707–17. 10.1016/j.jacc.2019.12.031 32081278

[34] Yang X Liang F Li J Chen J Liu F Huang K. Associations of long-term exposure to ambient PM2.5 with mortality in Chinese adults: a pooled analysis of cohorts in the China-PAR project. Environ. Int. (2020) 138:10558912. 10.1016/j.envint.2020.105589 32146266 PMC8164211

[35] Zheng C Tang H Wang X Chen Z Zhang L Kang Y. Left ventricular diastolic dysfunction and cardiovascular disease in different ambient air pollution conditions: a prospective cohort study. Sci Total Environ. (2022) 831:154872. 10.1016/j.scitotenv.2022.154872 35358529

[36] Nystoriak MA Bhatnagar A. Cardiovascular effects and benefits of exercise. Front Cardiovasc Med. (2018) 5:135. 10.3389/fcvm.2018.00135 30324108 PMC6172294

[37] Tucker WJ Fegers-Wustrow I Halle M Haykowsky M Chung EH Kovacic J. Exercise for primary and secondary prevention of cardiovascular disease: JACC focus seminar 1/4. J Am Coll Cardiol. (2022) 80:1091–106. 10.1016/j.jacc.2022.07.004 36075680

[38] Mora S Cook N Buring JE Ridker PM Lee I-M. Physical activity and reduced risk of cardiovascular events: potential mediating mechanisms. Circulation. (2007) 116:2110–8. 10.1161/CIRCULATIONAHA.107.729939 17967770 PMC2117381

[39] Boehm JK Kubzansky LD. The heart's content: The association between positive psychological well-being and cardiovascular health. Psychol Bull. (2012) 138:655–91. 10.1037/a0027448 22506752

[40] Havranek EP Mujahid MS Barr DA Blair IV Cohen MS Cruz-Flores S. Social determinants of risk and outcomes for cardiovascular disease: a scientific statement from the American Heart Association. Circulation. (2015) 132:873–98. 10.1161/CIR.0000000000000228 26240271

