# Supplementary material for: Blue sky as a protective factor for cardiovascular disease
Source: Front Public Health. 2022 Oct 14;10:1016853. doi: 10.3389/fpubh.2022.1016853 (PMC9614020; doi:10.3389/fpubh.2022.1016853)
Supplement: Supplementary file 1 [file Table_1.DOCX]

**Blue sky as a protective factor for cardiovascular disease**

Haosu Tang^1,2,3,5#^, Congyi Zheng^2#^, Xue Cao^2#^, Su Wang^1,5^, Linfeng Zhang^2^, Xin Wang^2^, Zuo Chen^2^, Yuxin Song^2^, Chen Chen^2^, Yixin Tian^2^, Wenping Jiang^4^, Gang Huang^1,3,5*^, Zengwu Wang^2*^, for the China hypertension survey investigators

*^1^**State key Laboratory of Numerical Modeling for Atmospheric Sciences and Geophysical Fluid Dynamics (LASG) / Center for Monsoon System Research (CMSR), Institute of Atmospheric Physics, Chinese Academy of Sciences, Beijing, 100029, China*

*^2^Division of Prevention and Community Health, National Center for Cardiovascular Disease, National Clinical Research Center of Cardiovascular Disease, State Key Laboratory of Cardiovascular Disease, Fuwai Hospital, Peking Union Medical College & Chinese Academy of Medical Sciences, Beijing, 102308, China*

*^3^Laboratory for Regional Oceanography and Numerical Modeling, Qingdao National Laboratory for Marine Science and Technology, Qingdao, 266237, China*

*^4^Key Laboratory of Marine Hazards Forecasting, Ministry of Natural Resources / College of Oceanography, Hohai University, Nanjing, 210024, China*

*^5^University of Chinese Academy of Sciences, Beijing, 100049, China*

^#^These authors contributed equally.

^*^Corresponding to G.H. ([hg@mail.iap.ac.cn](mailto:hg@mail.iap.ac.cn)) or Z.W. ([wangzengwu@foxmail.com](mailto:wangzengwu@foxmail.com))

**Contents**

- Supplemental Figures S1-2
- Supplemental Tables S1-3
- Supplemental Texts S1


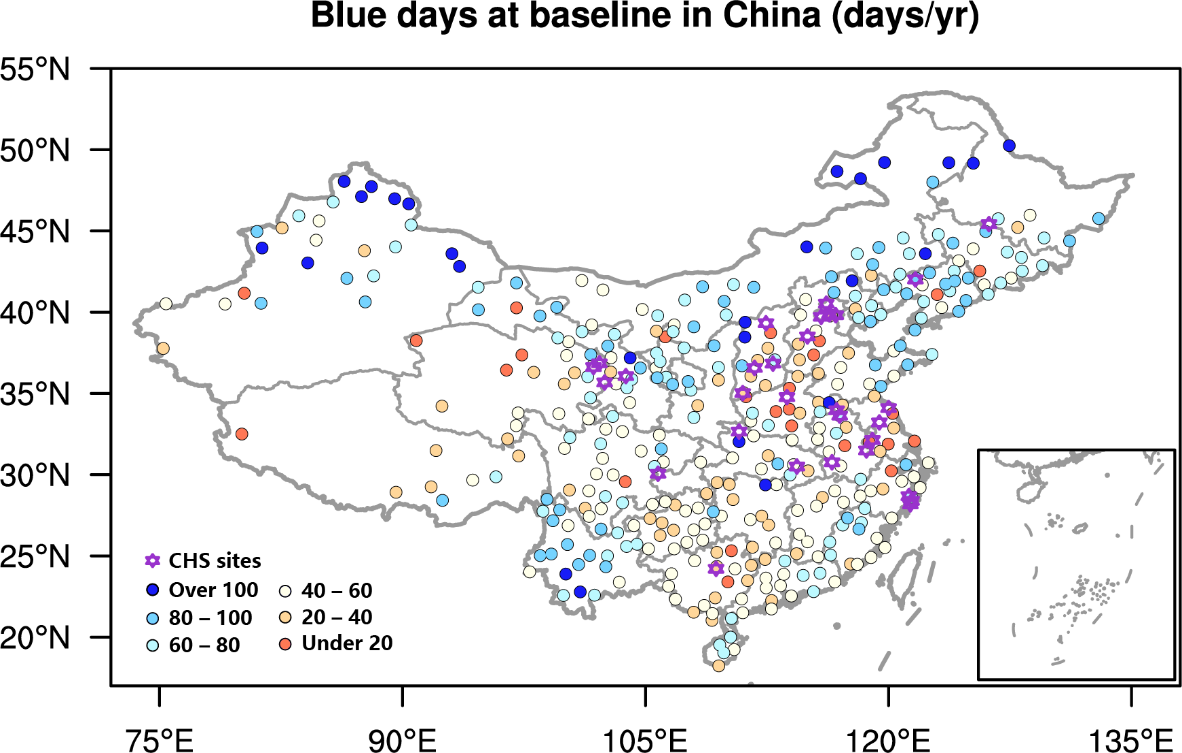


**Figure S1.** The geographical distributions of 378 ground meteorological stations across China (dot colors indicate annual blue day exposure at baseline, days/year). Purple stars denote 30 China Hypertension Survey sites.


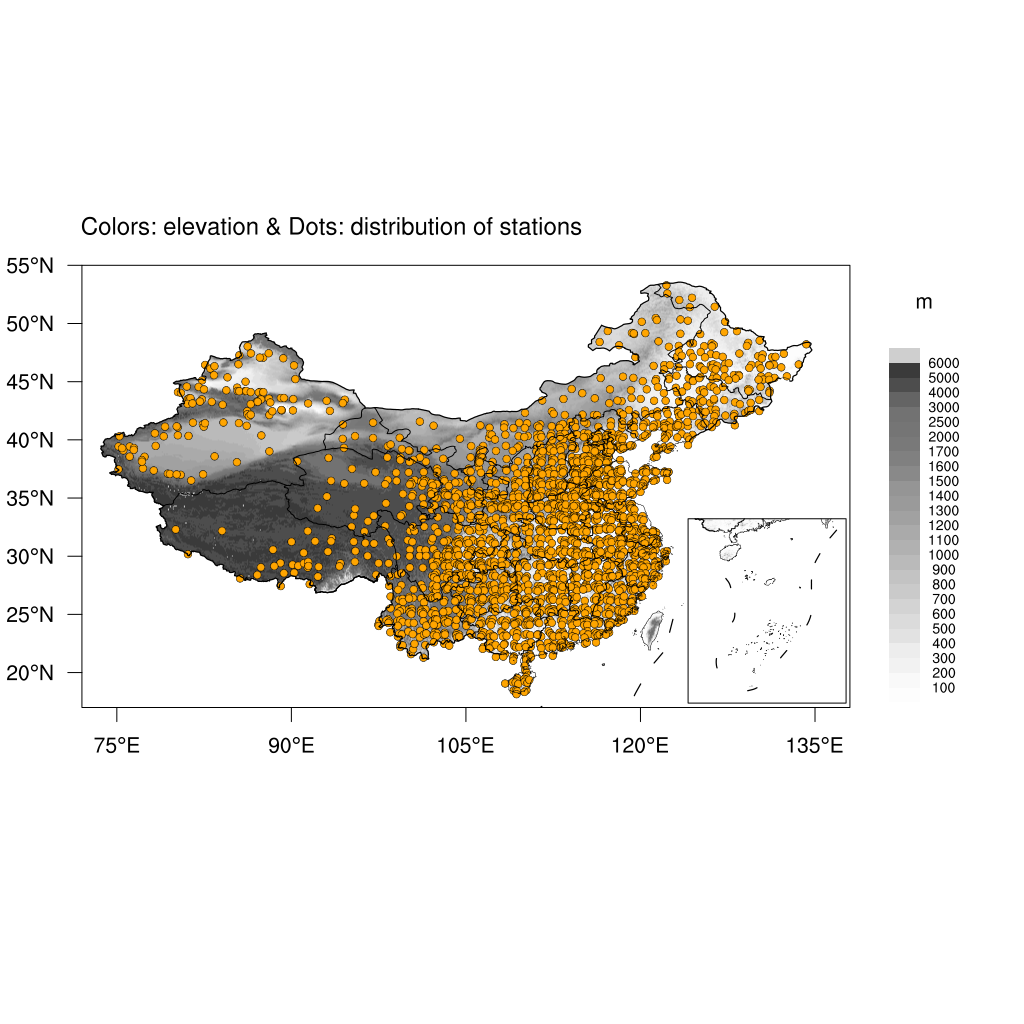


**Figure S2.** Distribution of ~2419 meteorological stations in China (orange points), superimposed on the elevation (shading; m). Inset: South China Sea.

**Table S1.** Baseline characteristics of the participants included in this study and the original population.

| **Characteristics** | **Included** | **Original** | ***P* value** |
| --- | --- | --- | --- |
| Number of participants | 22,702 | 30,036 | / |
| Age, years | 56.1±13.1 | 57.0±13.3 | <0.001 |
| Male | 10,505 (46.3) | 13,872 (46.2) | <0.001 |
| Urban | 10,130 (44.6) | 14,943 (49.8) | <0.001 |
| Han ethnicity | 20,315 (89.5) | 27,134 (90.3) | <0.001 |
| Region |  |  |  |
| East | 9,263 (40.8) | 12,526 (41.7) | <0.001 |
| Central | 9,460 (41.7) | 12,122 (40.4) | <0.001 |
| West | 3,979 (17.5) | 5,388 (17.9) | <0.001 |
| Education at least middle school | 11,109 (48.9) | 15,303 (50.9) | <0.001 |
| Smoking |  |  |  |
| Current | 5,731 (25.2) | 7,305 (24.3) | <0.001 |
| Former | 1,216 (5.4) | 1,927 (6.4) | <0.001 |
| Never | 15,755 (69.4) | 20,804 (69.3) | <0.001 |
| Alcohol consumption | 6,318 (27.8) | 8,235 (27.4) | <0.001 |
| BMI (kg/m^2^) |  |  |  |
| Normal | 10,213 (45.0) | 13,401 (44.6) | <0.001 |
| Overweight | 8,533 (37.6) | 10,809 (36.0) | <0.001 |
| Obesity | 3,956 (17.4) | 5,826 (19.4) | <0.001 |
| Hypertension | 8,957 (39.5) | 12,501 (41.6) | <0.001 |
| Hypercholesterolemia | 7,724 (34.0) | 10,544 (35.1) | <0.001 |
| Diabetes mellitus | 2,286 (10.1) | 3,436 (11.4) | 0.201 |
| Family history of CVD | 2,621 (11.5) | 4,663 (15.5) | <0.001 |
| CVD medication history | 4,929 (21.7) | 6,637 (22.1) | <0.001 |
| BMI, body mass index; SD, standard deviation. CVD, cardiovascular disease.  Data are represented as mean ± standard deviation or number (%). | | | |

**Table S2.** Stratified numbers of baseline annual blue days.

|  | Number of participants, no. (%) | Annual blue days (days/year) |
| --- | --- | --- |
| Gender |  |  |
| Male | 10,505 (46.3) | 57.55 |
| Female | 12,197 (53.7) | 56.34 |
| Urbanity |  |  |
| Urban | 10,130 (44.6) | 53.91 |
| Rural | 12,572 (55.4) | 59.31 |
| Region |  |  |
| East | 9,263 (40.8) | 55.37 |
| Central | 9,460 (41.7) | 55.32 |
| West | 3,979 (17.5) | 64.22 |
| PM_2.5_, μg/m^3^ |  |  |
| High (≥ 67.94) | 7,325 (32.3) | 53.39 |
| Middle (51.97–67.94) | 7,475 (32.9) | 49.44 |
| Low (≤ 51.97) | 7,902 (34.8) | 67.21 |
| NO_2_, μg/m^3^ |  |  |
| High (≥ 31.40) | 7,605 (33.5) | 44.79 |
| Middle (22.76–31.40) | 7,972 (35.1) | 68.55 |
| Low (≤ 22.76) | 7,125 (31.4) | 56.79 |
| O_3_, μg/m^3^ |  |  |
| High (≥ 61.06) | 8132 (35.8) | 57.16 |
| Middle (52.09–61.06) | 6667 (29.4) | 62.05 |
| Low (≤ 52.09) | 7903 (34.8) | 52.29 |
| Ambient temperature,°C |  |  |
| High (≥ 27.35) | 7287 (32.1) | 41.08 |
| Middle (23.46–27.35) | 7279 (32.1) | 60.65 |
| Low (≤ 23.46) | 8136 (35.8) | 67.71 |
| Relative Humidity, % |  |  |
| High (≥ 71.45) | 7246 (31.9) | 55.67 |
| Middle (62.50–71.45) | 7817 (34.4) | 57.53 |
| Low (≤ 62.50) | 7639 (33.7) | 57.42 |
| Abbreviations: PM_2.5_, particles with an aerodynamic diameter of ≤ 2.5 μm; NO_2_, nitrogen dioxide; O_3_, ozone. | | |

**Table S3.** Sensitivity analyses for hazard ratios (95% CIs) of fatal or nonfatal CVD and stroke with the best tertile compared to the worst tertile in annual blue day exposure.

| Models | Fatal or nonfatal CVD | Fatal or nonfatal stroke |
| --- | --- | --- |
| raw | 0.68 (0.57−0.81) | 0.57 (0.45−0.71) |
| a. fixed threshold definition | 0.66 (0.54−0.82) | 0.54 (0.41−0.71) |
| b. 3-year mean before baseline | 0.71 (0.59−0.86) | 0.58 (0.45−0.74) |
| c. dichotomous PM_2.5_ | 0.67 (0.56−0.79) | 0.55 (0.44−0.69) |
| d. excluding subjects with early deaths | 0.65 (0.54−0.79) | 0.56 (0.44−0.72) |
| e. excluding subjects with residential movement | 0.64 (0.52−0.78) | 0.53 (0.40−0.68) |
| f. excluding subjects with baseline diseases | 0.71 (0.58−0.85) | 0.59 (0.46−0.73) |
| g. Model 4 + relative humidity | 0.67 (0.57−0.81) | 0.56 (0.45−0.71) |
| h. Model 4 + heat wave frequency | 0.73 (0.59−0.90) | 0.60 (0.46−0.79) |
| i. Model 4 + NDVI | 0.68 (0.57−0.81) | 0.60 (0.45−0.72) |
| i. Model 4 + EVI | 0.74 (0.61−0.88) | 0.64 (0.50−0.80) |
| j. Model 4 + NO_2_ | 0.69 (0.58−0.82) | 0.56 (0.45−0.71) |
| j. Model 4 + O_3_ | 0.77 (0.64−0.92) | 0.63 (0.49−0.79) |
| Abbreviations: CVD, cardiovascular disease; PM_2.5_, particles with an aerodynamic diameter of ≤ 2.5 μm; NDVI, normalized difference vegetation index; EVI, enhanced vegetation index; NO_2_, nitrogen dioxide; O_3_, ozone. | | |

**Text S1.** List of the China Hypertension Survey Investigators.

This study could not be accomplished without the fine work of the staff at the national level. For a partial listing of colleagues see the follows (provinces sorted as alphabetical order):

**Anhui:** Liqun Hu, Hongqi Li, Qi Zhang, Guang Yan, Anhui Provincial Hospital, Hefei, Anhui, China; Fangfang Zhu, Anhui Institute of Cardiovascular Disease, Hefei, Anhui, China.

**Beijing:** Xianghua Fang, Chunxiu Wang, Shaochen Guan, Xiaoguang Wu, Hongjun Liu, Chengbei Hou, Xuanwu Hospital, Capital Medical University, Beijing, China.

**Chongqing:** Han Lei, Wei Huang, Nan Zhang, First Affiliated Hospital of Chongqing Medical University, Chongqing, China; Ge Li, Lihong Mu, Xiaojun Tang, Chongqing Medical University, Chongqing, China.

**Fujian:** Ying Han, Huajun Wang, Dongjie Lin Liangdi Xie, First Affiliated Hospital of Fujian Medical University, Fuzhou, Fujian, China; Daixi Lin, Fujian medical university, Fuzhou, Fujian, China.

**Gansu:** Jing Yu, Xiaowei Zhang, Wei Liang, Heng Yu, Qiongying Wang, Lanzhou University Second Hospital, Lanzhou, Gansu, China; Lan Yang, Maternal and Child Care Service Centre, Lanzhou, Gansu, China.

**Guangdong:** Yingqing Feng, Yuqing Huang, Guangdong General Hospital, Guangzhou, Guangdong, China; Peixi Wang, Jiaji Wang, Guangzhou Medical University, Guangzhou, Guangdong, China; Harry HX Wang, Sun Yat-Sen University, Guangzhou, Guangdong, China; Songtao Tang, Community Health Services Center of Liaobu, Dongguan, Guangdong, China.

**Guangxi:** Tangwei Liu, Rongjie Huang, Zhiyuan Jiang, Haichan Qin, First Affiliated Hospital of Guangxi Medical University, Nanning, Guangxi, China.

**Guizhou:** Guoqin Liu, Zhijun Liu, Wenbo Rao, Zhen Chen, Yalin Chu, Fang Wu, Zunyi Medical University, Zunyi, Gouzhou, China.

**Hainan:** Haitao Li, Jianlin Ma, Tao Chen, Hainan General Hospital, Haikou, Hainan, China; Ming Wu, Health and Family Planning Commission of Hainan, Haikou, Hainan, China.

**Hebei:** Jixin Sun, Yajing Cao, Yuhuan Liu, Center for Disease Prevention and Control of Hebei, Shijiazhuang, Hebei, China; Zhikun Zhang, Center for Disease Prevention and Control of Tangshan, Tangshan, Hebei, China; Yanmei Liu, Center for Disease Prevention and Control of Langfang, Langfang, Hebei, China; Dejin Dong, Center for Disease Prevention and Control of Xingtai, Xingtai, Hebei, China; Guangrong Li, Center for Disease Prevention and Control of Dingzhou, Dingzhou, Hebei, China.

**Heilongjiang:** Hong Guo, Lihang Dong, Haiyu Zhang, Fengyu Sun, Xingbo Gu, Ye Tian, First Affiliated Hospital of Harbin Medical University, Haerbin, Heilongjiang, China.

**Henan:** Kaijuan Wang, Chunhua Song, Peng Wang, Hua Ye, Zhengzhou University, Zhengzhou, Henan, China; Wei Nie, Shuying Liang, Henan Academy of Medical Sciences, Zhengzhou, Henan, China.

**Hubei:** Congxin Huang, Fang Chen, Yan Zhang, Heng Zhou, Jing Xie, Jianfang Liu, Department of Cardiology, Renmin Hospital of Wuhan University, Wuhan, Hubei, China.

**Hunan:** Hong Yuan, Chengxian Guo, Third Xiangya Hospital, Central South University, Changsha, Hunan, China; Yuelong Huang, Biyun Chen, Center for Disease Control and Prevention of Hunan, Changsha, Hunan, China.

**Inner Mongolia:** Xingsheng Zhao, Wenshuai He, Xia Wen, Yanan Lu, Inner Mongolia people's hospital, Hohhot, Inner Mongolia, China.

**Jiangsu:** Xiangqing Kong, Ming Gui, Wenhua Xu, Yan Lu, Jun Huang, First Affiliated Hospital of Nanjing Medical University, Nanjing, Jiangsu, China; Min Pan, Affiliated Hospital of Nantong University, Nanjing, Jiangsu, China; Jinyi Zhou, Ming Wu, Center for Disease Control and Prevention of Jiangsu, Nanjing, Jiangsu, China.

**Jiangxi:** Xiaoshu Cheng, Huihui Bao, Xiao Huang, Kui Hong, Juxiang Li, Ping Li, Second Affiliated Hospital of Nanchang University, Nanchang, Jiangxi, China.

**Jilin:** Bin Liu, Junduo Wu, Longbo Li, Yunpeng Yu, Yihang Liu, Chao Qi, Second Hospital of Jilin University, Changchun, Jilin, China.

**Liaoning:** Jun Na, Li Liu, Yanxia Li, Guowei Pan, Center for Disease Prevention and Control of Liaoning, Shenyang, Liaoning, China; Degang Dong, Peng Qu, Health and Family Planning Commission of Liaoning, Shenyang, Liaoning, China.

**Ningxia:** Jinbao Ma, Health and Family Planning Commission of Ning Xia Hui Autonomous Region, Yinchuan, Ningxia, China; Juan Hui, Center for Disease Control and Prevention of Ning Xia Hui Autonomous Region, Yinchuan, Ningxia, China; Fu Zhao, Health Supervision Institute of Xixia District in Yinchuan, Ning Xia Hui Autonomous Region, Yinchuan, Ningxia, China.

**Qing Hai:** Jianning Yue, Minru Zhou, Zhihua Xu, Xiaoping Li, Qiongyue Sha, Fuchang Ma, Qing Hai Center for Disease Control and Prevention, Xining, Qinghai, China; Qiuhong Chen, Huiping Bian, Qinghai Cardio-Cerebrovascular Disease Special Hospital, Xining, Qinghai, China.

**Shaanxi:** Jianjun Mu, Tongshuai Guo, Keyu Ren, Chao Chu, First Affiliated Hospital of Xi’an Jiaotong University, Xian, Shaanxi, China.

**Shandong:** Zhendong Liu, Hua Zhang, Yutao Diao, Shangwen Sun, Yingxin Zhao, Institute of Basic Medicine, Shandong Academy of Medical Sciences, Jinan, Shandong, China.

**Shanghai:** Junbo Ge, Jingmin Zhou, Xuejuan Jin, Jun Zhou, Zhongshan Hospital, Fudan University, Shanghai, China.

**Shanxi:** Bao Li, Lijun Zhu, Yuean Zhang, Gang Wang, Shanxi Cardiovascular Hospital, Taiyuan, Shanxi, China; Zhihan Hao, Wuxiang County People's Hospital, Wuxiang, Shanxi, China.

**Sichuan:** Li Cai, Zhou Liu, Zhengping Yong, Jianhong Tao, Yijia Tang, Sichuan Provincial People's Hospital, Chengdu, Sichuan, China; Shaoping Wan, Sichuan Cancer Hospital, Chengdu, Sichuan, China.

**Tianjin:** Zhenshan Jiao, Yuqiang Fan, Tianjin Academy of Traditional Chinese Medicine, Tianjin, China; Hui Gao, Wei Wang, Tianjin Municipal Commission of Health and Family Planning, Tianjin, China; Qingkui Li, Xiaomei Zhou, Tianjin Medical University, Tianjin, China.

**Tibet:** Yundai Chen, Bin Feng, Qinglei Zhu, Sansan Zhou, Chinese People’s Liberation Army General Hospital, Lasha, Tibet, China.

**Xinjiang:** Nanfang Li, Lin Zhou, Delian Zhang, Jing Hong, People's Hospital of Xinjiang Uygur Autonomous Region, Urumuqi, Xinjiang, China.

**Yunnan:** Tao Guo, Min Zhang, First Affiliated Hospital of Kunming Medical University, Kunming, Yunnan, China; Yize Xiao, Center for Disease Prevention and Control of Yunnan, Kunming, Yunnan, China; Xuefeng Guang, Affiliated Yan'an Hospital of Kunming Medical University, Kunming, Yunnan, China.

**Zhejiang:** Xinhua Tang, Jing Yan, Xiaoling Xu, Li Yang, Aimin Jiang, Wei Yu, Zhejiang Hospital, Hangzhou, Zhejiang, China.
